# Establishment of a physical exercise evaluation index system for school-age children with asthma

**DOI:** 10.1371/journal.pone.0312398

**Published:** 2025-01-09

**Authors:** Yueheng Zhao, Xianmei Meng, Suqing Wang

**Affiliations:** 1 School of Nursing, Wuhan University, Wuhan, China; 2 Sichuan Vocational College of Health and Rehabilitation, Zigong, China; Bindura University of Science Education, SOUTH AFRICA

## Abstract

**Objective:**

To construct a comprehensive physical exercise evaluation index system for asthmatic children aged 6–12 years.

**Design:**

Based on knowledge-attitude-practice(KAP) theory, we constructed an item pool for a physical exercise evaluation index system using a literature review and semistructured interviews and refined the index system through two questionnaire cycles with Delphi experts.

**Results:**

For the two questionnaire rounds, the recovery rate was 100%, the experts’ authority coefficients were 0.850 and 0.836, and the coordinated coefficients were 0.167 and 0.202 (P<0.001). Finally, four first-level indicators, namely, disease factors, exercise environment, exercise practice, and exercise psychology; 11 second-level indicators; and 50 third-level indicators were developed as a physical exercise evaluation index system for asthmatic children.

**Conclusion:**

A physical exercise evaluation index system built using a literature review, qualitative interviews, and Delphi technique can provide scientific guidance for children with asthma, caregivers and health professionals to comprehensively assess the children’s physical exercise.

## Introduction

Asthma is one the most common chronic respiratory disease and affects up to 18% of the population in some countries [1]. Typically, patients exhibit recurrent respiratory symptoms such as dyspnea and cough, which seriously affect their quality of life. According to statistics from the World Health Organization, in 2019, 262 million people were affected by asthma, and 455,000 people died [2].The prevalence of asthma among Chinese children was 4.90% [3]. The school age is from the beginning of primary school for 6–7 years old to pre-adolescence (generally 11–12 years old) [4]. One of the most common diseases in school-age children is asthma that has the characteristics of long course of disease and easy recurrence [5], which affects the physical and mental health of children.

Among the many triggers of asthma, exercise is a special presence. Studies have shown that exercise may induce or even aggravate asthma under certain circumstances [6, 7]. However, research in recent years has demonstrated that appropriate regular exercise can play a positive role in the management of asthma. Exercise intervention is safer and more economic than drug therapy [8]. The Global Strategy for Asthma Management and Prevention also states that regular and moderate physical activity is beneficial to the health of people with asthma [9]. The Australian Association for Exercise and Sports Science suggests that patients with asthma should participate in sports activities and exercise allows people with asthma to live as normal a physical lifestyle as possible, and with pre-exercise medication, most people with asthma can participate in exercise on an equal footing with non-asthmatic people of the same size and fitness level [10]. Children with asthma should take regular exercise, exercise can improve children’s lung function index, immune system, exercise ability, and improve quality of life [11]. Aerobic exercise is beneficial for both acute and chronic inflammatory asthma [12]. Exercise can increase forced expiratory volume in the first second (FEV_1_) and forced lung capacity, and improve lung function in children [13]. Children with asthma who engage in regular physical activity have a lower rate of hospitalization [14]. Physical exercise can significantly improve FVC in children [15]. And under proper management and monitoring, moderate intensity aerobic exercise has good safety and effectiveness, and can improve the exercise ability and quality of life of children with asthma [16].

Asthma continues to affect the daily life of children at school, including negative effects on their attendance and physical education by the disease and its symptoms [17]. Exercise can trigger asthma under adverse conditions, but if health care professionals can guide children to exercise properly, exercise can change from a trigger to a beneficial factor. Therefore, children with asthma should perform physical exercise and may even be prescribed physical exercise as a treatment [18]. But literature and clinical investigations suggest that exercise can trigger asthma in some cases [19], and children and their parents may face substantial psychological pressure and worry about aggravating the disease [20]. Some patients accept physical exercise, but for disease management, the choice of exercise environment and exercise practice is unreasonable and may increase the risk of asthma attack.

There are recommendations and prescriptions for exercise for children with asthma, for example, the Global Strategy for Asthma Management and Prevention [9]; the American College of Sports Medicine (ACSM) Guidelines for exercise testing and prescription [21]; and expert consensus on exercise prescription for asthmatic children in China [22]. Questionnaires are available to evaluate physical activity levels from childhood to adolescence and the Physical Activity Questionnaire for Older Children (PAQ-C) and Adolescents (PAQ-A) Manual) [23]. However, at present, there is no exercise evaluation index system for patients with asthma in China or abroad, despite the high prevalence of asthma among children and need for exercise. Therefore, our study aimed to develop a set of indicators of a physical exercise evaluation system for school-aged children with asthma to provide a scientific tool for both children with asthma, their caregivers and hospital professionals to assess the physical exercise of children with asthma, which may serve as a basis for guiding the exercise management of children with asthma and reducing their risk of exercise-induced asthma (EIA).

## Material and methods

### Constructing the primary physical exercise evaluation index system for school-age children with asthma

#### Literature review

The method of combining the topic and free words was used for retrieval. The retrieval words were bronchial asthma or asthma, exercise or physical exercise, evaluation system or assessment systems, evaluation indicators and systematic construction. The main search databases used were the Global Initiative for Asthma (GINA), Web of Science, PubMed/Medline, China National Knowledge Infrastructure(CNKI) and China Science and Technology Journal Database. In addition, a two-month field study in the pediatric asthma clinic was conducted to collect information about physical exercise in children with asthma. Knowledge, Attitude and Practice Theory(KAP theory):The theory that beliefs are influenced by knowledge and that attitude change behavior [24].This study explored the influencing factors of exercise in asthmatic children from the perspectives of knowledge, belief and behavior. To further construct the physical activity evaluation index system of school-age children with asthma. To establish measurable indicators, through the assessment of pediatric asthma specialist medical staff, timely find the shortcomings, establish a targeted education and guidance mechanism, and improve the exercise level of children with asthma.

#### Qualitative interview

Five experts were selected by objective sampling to conduct semi-structured interviews. The expert inclusion criteria were as follows: ① being a pediatric nurse, respiratory nurse, medical expert, rehabilitation or community nursing expert providing medical, nursing care or rehabilitation services for asthma patients for 5 years or longer; ② having an intermediate or senior professional title; ③ having a bachelor’s degree or above in nursing or clinical medicine; and ④ consent to participate. Through literature review and interviews, finally a preliminary 3-level evaluation index system was developed, including 4 first-level indicators, 13 second-level indicators, and 39 third-level indicators.

#### Two-round Delphi survey

Refer to semi-structured interview expert selection requirements, ultimately, 20 experts participated and completed two rounds of the Delphi survey. Among them, 11 experts were from the respiratory and pediatric departments of two tertiary general hospitals; 4 experts were from university nursing schools; and 5 experts were from the clinical medical technology department and vocational college nursing schools. These experts were between 36 and 57 years old (average age of 46 years) and had 13–39 years of work experience (average work experience of 24.6 years); 5 were doctors, 15 were nurses, 11 had undergraduate degrees, 7 had master’s degrees, and 2 had doctoral degrees.

The questionnaire included the research background, research purpose, system construction basis and introduction, completion instructions, text and expert basic information. The experts scored the first, second and third-level indicators in the questionnaire on a 5-point Likert scale (very important, important, moderately important, not very important, and not important), with scores ranging from 1–5 points. Moreover, experts proposed their own suggestions for modifying the items (S1 Table).

There were two rounds of Delphi expert consultation in this study. In the first round consultation, all 20 experts returned the questionnaires. Indicators with a mean importance score greater than 3.5, a full score ratio > 0.2, and a variation coefficient <0.25 were included in the second-round survey [25]. In addition, a research team with three members discussed the opinions, suggestions, and other expert feedback carefully and revised or eliminated items accordingly. Finally, 4 first-level indicators, 11 second-level indicators and 50 third-level indicators were constructed. In the second round, 20 experts scored the indicators of the system again on a 5-point Likert scale and proposed suggestions for modification (S2 Table). None of the 20 experts raised new objections. All the indicators satisfied an average importance score greater than 3.5, a perfect score ratio > 0.2, and a coefficient of variation <0.25; thus, they were retained.

WPS2019 and SPSS 20.0 software were used for statistical analysis of the data. The mean importance score, full score ratio, coefficient of variation, variance, expert authority coefficient, degree of familiarity, judgment basis and Kendall’s W coefficient were calculated for the indicators. Analytic Hierarchy Process, referred to as AHP, is a decision-making method that decomposes the elements related to decision into levels such as target, criterion and schemes, and then carries out qualitative and quantitative analysis [26]. The analytic hierarchy process (AHP) was used in this study. The first step involved establishing a hierarchical structure model and determining the Satty standard on the basis of the differences in the importance of the indicators among experts. System includes target level, criterion level and program level. The second step involved building a judgment matrix and calculating the weights of the indicators at all levels.

#### Ethics statements

This study did not involve patient information and no patient intervention was performed. For the qualitative Interview and Delphi survey and consent was obtained from potential participants through the internet after the participants had received an adequate explanation of the study. The informed consent of all participating experts is in writing.

## Results

### Qualitative interview

Based on semi-structured interviews with 5 experts and literature review, four themes of the evaluation system were summarized. The basic information of the five interview experts is shown in Table 1.

**Table 1:** Basic information of interview experts(n = 5).

Code	Gender	Professional title	Years of working/research	Specialty
A	F	Associate Professor	14	Pediatric Nursing
B	F	Associate Professor	13	Pediatric Nursing
C	F	Associate Professor	30	Respiratory Therapy
D	F	Professor	31	Respiratory Nursing
E	F	Professor	28	Pediatric Nursing

#### Theme 1: Disease factors

The most important factor affecting the exercise of children with asthma is the disease. Experts A, C and E mentioned that the exercise level of asthma patients was positively correlated with the asthma control level. Expert B stressed that allergic substances and medication compliance should not be ignored.

#### Theme 2: Exercise environment

Experts B and E stressed that whether the exercise environment has allergens is the key, and the temperature and humidity of the air will also affect asthma symptoms. Expert C mentioned that in the stage of poor asthma control, whether the exercise has a guardian and relevant monitoring equipment is very important.

#### Theme 3: Exercise knowledge

Expert D stressed that exercise for asthmatic patients should follow the exercise prescription, and choose the right time, frequency and type of exercise.

#### Theme 4: Exercise psychology

Expert A mentioned that the negative perception of exercise by children or their families can affect the level of exercise. Expert D stressed that children or family members who recognize the positive effects of exercise on asthma control are key to improving exercise levels.

### Two-round Delphi survey

In the first round, a total of 20 experts participated in the consultation, with 20 questionnaires distributed and a questionnaire return rate of 100%. Ten experts proposed changes to the indicator system. In the second round, the questionnaire return rate was 100%, and no expert provided new advice. The expert authority coefficient was calculated as follows: (Cr) = (Ca+Cs)/2,Cr based on Ca and Cs assignments (S3 Data). It is generally believed that when Cr ≥ 0.7, the results of expert consultation are reliable [27], and the greater the Cr is, the greater the reliability. The degree of expert authority of the two Delphi rounds was >0.7, indicating that the authority of the experts was high. The results of the two rounds of Delphi correspondence are shown in Tables 2–8 (S1, S2 Data).

**Table 2:** Basic information of Delphi experts.

Content	Categories	First/Second round(n = 20)	
		Number of people	Percentage
Gender	F	16	80%
	M	4	20%
Age	30~	3	15%
	40~	10	50%
	50~	7	35%
Education	Undergraduate	11	55%
	Graduate	7	35%
	Doctor	2	10%
Professional title	Medium-grade	2	10%
	Associate Professor	14	70%
	Professor	4	20%
Specialty	Pediatric Nursing	7	35%
	Respiratory Nursing	8	40%
	Pediatric Therapy	1	5%
	Respiratory Therapy	4	20%
Years of working/research	10~	6	30%
	20~	8	40%
	30~	6	30%

**Table 3:** Expert authority.

Delphi round	Ca	Cs	Cr
	Judgment coefficient	Familiarity coefficient	Authority coefficient
First	0.895	0.805	0.850
Second	0.906	0.765	0.836

**Table 4:** Concentration of expert opinion.

Items	Inquiries in the first round			Inquiries in the second round		
	Mean importance assignment (Mj)	Full score ratio (Kj)	Coefficient of variation (CV)	Mean importance assignment (Mj)	Full score ratio (Kj)	Coefficient of variation (CV)
First-level indicator	4.450–4.800	0.500–0.800	0.085–0.154	4.450–4.900	0.550–0.900	0.063–0.154
Second-level indicator	4.350–4.900	0.450–0.900	0.063–0.201	4.250–4.850	0.400–0.850	0.076–0.200
Third-level indicator	3.850–4.850	0.200–0.850	0.076–0.242	4.050–5.000	0.350–1.000	0.000–0.213

Mj:the arithmetic average of all experts on indicator j

Kj:The ratio of the number of full marks obtained by indicator j to the total number of experts

CV:(Standard deviation/average) × 100%.

**Table 5:** Degree of coordination for two rounds of expert opinions.

Delphi round	Entry number	W	χ^2^	df	P
First	56	0.167	184.233	55	<0.001
Second	65	0.202	258.379	64	<0.001

W:Coordination coefficient

df:Degree of freedom.

**Table 6:** Amendments proposed.

Original indicators	Modified indicators
"exercise knowledge"	"exercise practice"
"first aid measures"	"first aid knowledge and skills"
"exercise content"	"exercise items"
"exercise intensity	Combined into "exercise plan"
exercise duration	
exercise frequency"	
"frequency of asthma symptoms during the day"	"whether the frequency of daytime asthma symptoms is ≤ 2 times/week"
"whether PEF/FEV1 is normal"	"whether the FEV1 value is > 80%"
"frequency of drug use"	"whether alleviating drugs or first aid treatment is needed"
"whether the role of the drug is known"	"whether the main role of the drug used is known"
"whether the correct medication method has been mastered"	"whether how to administer the drugs is known"
	“whether the method for drug inhalation has been mastered”
"whether compliance to the medication is high"	"whether the physician-directed regular quantitative medication regiment is followed"
"Whether the environment is warm"	"whether the temperature is considered and whether a time when the temperature is comfortable is chosen"
"whether the environment is moist"	"whether the humidity is considered and whether a time when the humidity is comfortable is chosen"
"whether the haze levels are low"	"whether attention is given to the degree of haze as identified by mobile phone and whether outdoor sports are chosen only when the level is low"
"whether there are no allergens in the environment"	"whether the exercise environment is evaluated for allergens"
"whether an exhalation peak flow velocity meter is carried"	"whether the exhalation peak flow velocity instrument is carried to predict an acute attack"
"whether a heart rate monitor is worn"	"whether a heart rate monitor is worn to monitor activity"
"whether there are guardians"	"whether there are adult guardians present during exercise"
"Whether there is monitoring sports equipment”	“whether treadmills that can monitor exercise time and intensity-monitoring equipment are used”
“whether the PEFR> 80%”	“whether general physical activity causes difficulty breathing"
"whether there are guardians who provide support"	"whether the guardian’s opinion is accepted regarding exercise"
"whether team movement is sought"	"whether the individual participates in collective movement happily”
“whether the individual is worried about exercise exacerbating asthma”	“whether there are worries about exercise affecting the treatment effect”
12 items were added to the third-level indicators:	“Whether there are clear triggers”
	“Whether there are any other coexisting respiratory system diseases”
	“Whether the time, frequency and contraindications of oral medication are known”
	“Whether the preparation and use of inhalation devices has been mastered”
	“Whether the ability to use inhalation drugs has been mastered”
	“Whether the signs of an acute asthma exacerbation can be “Whether the signs of an exacerbation of acute attacks can be identified”
	“Whether there is a cooldown activity after exercising (15 min)"
	"Whether the heartbeat is normal without chest pain"
	"Whether an exercise schedule is made in advance"
	"Whether exercise logs are recorded after exercise"
	"Whether exercise plans are considered favorable"
	"Whether there is confidence in adhering to the exercise plan"
1 item was deleted from the third-level indicators:	"Whether there is a psychological burden of worrying about asthma attacks"

**Table 7:** A physical exercise evaluation index system for school-age children with asthma.

First-level indicator	Second-level indicator	Third-level indicator
A Disease Factors	A1 Disease Diagnosis	A1.1 Whether there are clear allergens
		A1.2 Whether motor asthma or motor bronchial spasm has been diagnosed
		A1.3 Whether there are clear triggers
		A1.4 Whether there are any other coexisting respiratory system diseases
	A2 Asthma control level	A2.1 Whether the frequency of daytime asthma symptoms is ≤ 2 times/week
		A2.2 No nighttime asthma symptoms or waking up with choking
		A2.3 Whether the activity is unrestricted
		A2.4 Whether alleviating drugs or first aid treatment is needed
		A2.5 Whether the FEV1 value is > 80%
	A3 Medication	A3.1 Whether the main role of the drug used is known
		A3.2 Whether how to use the drugs is known
		A3.3 Whether the time, frequency and contraindications of oral medication are known
		A3.4 Whether the preparation and use of inhalation devices have been mastered
		A3.5 Whether the ability to use inhalation drugs has been mastered
		A3.6 Whether the physician-directed regular quantitative medication schedule is followed
	A4 First aid knowledge and skills	A4.1 Whether the signs of an impending asthma attack are recognized
		A4.2 Whether the signs of an acute asthma exacerbation can be identified
		A4.3 Whether emergency asthma medication is carried
		A4.4 Whether the peak expiratory flow rate meter can be used correctly
		A4.5 Whether there is awareness of the self-care measures for acute attacks
B Exercise environment	B1 Natural environment	B1.1 Whether the temperature is considered and whether the time when the temperature is comfortable is chosen
		B1.2 Whether the humidity is considered and whether the time when the humidity is comfortable is chosen
		B1.3 Whether attention is paid to the degree of haze as identified using mobile phones and whether outdoor sports are chosen only when the level is low
		B1.4 Whether the exercise environment is evaluated for allergens
	B2 Custodial factors	B2.1 Whether the exhalation peak flow velocity instrument is carried to predict an acute attack
		B2.2 Whether a heart rate monitor is worn to monitor activity
		B2.3 Whether there are adult guardians present during exercise
		B2.4 Whether treadmills that can monitor exercise time and intensity-monitoring equipment are used
C Exercise Practice	C1 Exercise items	C1.1 Whether there are warm-up preparation activities (15 min)
		C1.2 Whether there is a cooldown activity after exercising (15 min)
		C1.3 Whether there is a combination of aerobic exercise, resistance exercise, and flexibility exercise
		C1.4 Whether pursed-lip breathing and abdominal breathing are performed
	C2 Exercise status	C2.1 Whether exercise is avoided on an empty stomach and immediately after a meal
		C2.2 Whether general physical activity causes difficulty breathing
		C2.3 Whether the heartbeat is normal without chest pain
	C3 Exercise plan	C3.1 Whether low-intensity movement is combined with moderate-intensity movement
		C3.2 Whether an exercise schedule is made in advance
		C3.3 Whether exercise logs are recorded after exercise
		C3.4 Whether the time is limited (20 ~ 60 min/d)
		C3.5 Whether regular exercise is performed an average of 3–5 days per week
D Exercise Psychology	D1 Positive psychology	D1.1 Loves exercise or not
		D1.2 Whether the individual is convinced of the positive effects of exercise on asthma control
		D1.3 Whether the guardian’s opinion is accepted regarding exercise
		D1.4 Whether exercise plans are considered favorable
		D1.5 Whether there is confidence in adhering to the exercise plan
		D1.6 Whether the individual participates in collective movement happily
		D1.7 Whether exercise is an enjoyable experience
		D1.8 Whether the individual can respond to exercise-induced asthma
	D2 Negative psychology	D2.1 Whether there is fear of an asthma attack during exercise
		D2.2 Whether there are worries about exercise affecting the treatment effect

**Table 8:** The mean importance assignments, full score ratios, coefficients of variation, and weights of the indicators.

Indicators	Mean importance assignment	Full score ratio	Coefficient of variation	Weight
A	4.900	0.9	0.063	26.34%
B	4.650	0.7	0.126	25.00%
C	4.600	0.65	0.130	24.73%
D	4.450	0.55	0.154	23.93%
A1	4.750	0.75	0.094	6.64%
A2	4.850	0.85	0.076	6.78%
A3	4.700	0.7	0.100	6.57%
A4	4.550	0.55	0.112	6.36%
B1	4.650	0.65	0.105	12.85%
B2	4.400	0.45	0.136	12.15%
C1	4.500	0.55	0.135	8.27%
C2	4.300	0.4	0.153	7.91%
C3	4.650	0.65	0.105	8.55%
D1	4.600	0.65	0.130	15.38%
D2	4.250	0.45	0.200	8.54%
A1.1	4.700	0.8	0.140	1.62%
A1.2	4.950	0.95	0.045	1.70%
A1.3	4.950	0.95	0.045	1.70%
A1.4	4.700	0.7	0.100	1.62%
A2.1	4.650	0.65	0.105	1.35%
A2.2	4.550	0.6	0.133	1.32%
A2.3	4.700	0.7	0.100	1.36%
A2.4	4.700	0.7	0.100	1.36%
A2.5	4.750	0.75	0.094	1.38%
A3.1	4.500	0.6	0.153	1.02%
A3.2	4.900	0.9	0.063	1.11%
A3.3	4.700	0.7	0.100	1.06%
A3.4	4.950	0.95	0.045	1.12%
A3.5	5.000	1	0.000	1.13%
A3.6	4.950	0.95	0.045	1.12%
A4.1	4.750	0.75	0.094	1.27%
A4.2	4.750	0.75	0.094	1.27%
A4.3	4.950	0.95	0.045	1.33%
A4.4	4.500	0.6	0.153	1.20%
A4.5	4.800	0.8	0.085	1.29%
B1.1	4.300	0.4	0.153	3.18%
B1.2	4.250	0.4	0.169	3.15%
B1.3	4.150	0.4	0.196	3.07%
B1.4	4.650	0.7	0.126	3.44%
B2.1	4.300	0.5	0.186	3.07%
B2.2	4.200	0.45	0.213	3.00%
B2.3	4.450	0.6	0.171	3.18%
B2.4	4.050	0.35	0.204	2.90%
C1.1	4.400	0.5	0.155	2.08%
C1.2	4.300	0.4	0.153	2.03%
C1.3	4.350	0.5	0.171	2.06%
C1.4	4.450	0.6	0.171	2.10%
C2.1	4.600	0.65	0.130	2.67%
C2.2	4.500	0.55	0.135	2.62%
C2.3	4.500	0.6	0.153	2.62%
C3.1	4.450	0.65	0.199	1.76%
C3.2	4.300	0.5	0.186	1.70%
C3.3	4.250	0.5	0.200	1.68%
C3.4	4.300	0.5	0.186	1.70%
C3.5	4.350	0.55	0.187	1.72%
D1.1	4.150	0.45	0.211	1.44%
D1.2	4.750	0.75	0.094	1.65%
D1.3	4.450	0.55	0.154	1.54%
D1.4	4.450	0.6	0.171	1.54%
D1.5	4.700	0.75	0.122	1.63%
D1.6	4.300	0.45	0.170	1.49%
D1.7	4.450	0.65	0.186	1.54%
D1.8	4.600	0.7	0.148	1.60%
D2.1	4.500	0.65	0.169	5.81%
D2.2	4.400	0.55	0.171	5.68%

The degree of coordination of expert opinions reflects the degree of agreement among experts and is expressed as the coefficient of variation (CV) and Kendall’s W coefficient (W),and the smaller the CV value, the larger the W value, and the higher the expert coordination consistency [28] (Tables 5, 8).

According to the results of the two rounds of the Delphi survey and the research team discussion, the initial evaluation indicator system was amended. In the first round of expert consultation, the rate of the experts’ opinions was high, with a total of 35 amendments proposed (Table 6). Finally, 4 first-level indicators, 11 second-level indicators, and 50 third-level indicators were identified, among them, the target level is to build a sports evaluation system for school-age children, the criterion level is the first and second indexes, and the program level is the third indexes (Table 7). The importance of the indicators at all levels, the full score ratios, the mutation coefficients and the weighted results are presented in Table 8.

## Discussion

### Content analysis of the physical exercise self-evaluation index system for school-age children with asthma

In our study, a set of exercise evaluation indicators for children with asthma, including 4 first-level indicators, 11 second-level indicators, and 50 third-level indicators, was developed. These indicators were grouped into four categories; disease, environment, exercise and psychology, and the factors that may trigger or exacerbate asthma and affect exercise quality in children with asthma were also included. The four first-level indicators were weighted evenly, indicating that the system was formulated reasonably. Among these factors, disease-related factors had the greatest weight, indicating that experts believe that the most important factor affecting exercise is the management of asthma. The secondary indicators are patient awareness of their disease diagnosis, asthma control level, medication, first aid knowledge and skills, with effectively balanced weights, and the asthma control level is recognized as the most influential factor. Among the environmental factors, the natural environment is considered more important than the custodial environment. Among the psychological factors, both positive and negative psychological conditions were considered, as a previous study revealed that psychological health influences behavior [29]. Positive psychological status generally helps to improve the effectiveness of practice; conversely, negative psychological status can hinder practice and development. The characteristic of this system is that the evaluation of exercise in children with asthma is not limited to exercise itself, but combines the three aspects of disease, environment and psychology to make a comprehensive evaluation, taking into account various factors affecting exercise in an all-round way, which is conducive to the evaluation of nursing staff and the establishment of scientific and systematic management of exercise in children with asthma.

### The scientific nature of the physical exercise evaluation index system for school-age children with asthma

According to the SMART principle(Specific, Measurable, Achievable, Relevant, Time-bound), the initial indicator formulation of the evaluation index system was based on a literature review; literature with Grade A evidence was preferentially selected, and the indicators were developed by combining semistructured clinical interviews. In addition, two rounds of expert correspondence consultations and an analytic hierarchy process were combined with qualitative and quantitative research to form a three-level evaluation indicator system and index weights. In addition, the Delphi expert consensus method was adopted to review and revise the preliminary evaluation index system. To collect comprehensive and scientific views and ideas, 20 experts from related disciplines, such as respiratory and pediatric nursing, medicine, nurse educators, and respiratory rehabilitation, were invited to participate. The Delphi survey was conducted for two rounds until the agreement among the experts was met and there were no further suggestions. The general number of experts required for the Delphi technique is 15–30. At present, there are many exercise prescriptions for children with asthma, but most of them start from the single dimension of exercise, and do not include the evaluation content of disease, environment and psychology. In contrast, the exercise evaluation of asthmatic children in this system is more comprehensive, and pays attention to the evaluation of exercise-related factors.

### Impact

In clinical practice, a safe and reasonable physical exercise evaluation index system for children with asthma is lacking, and school-age children have a lower physical exercise safety coefficient than adults do; thus, more guidance is needed. The establishment of a physical exercise evaluation index system for school-age children with asthma has practical clinical significance.

When children with asthma play sports, they are affected by the disease, the environment, exercise conditions and psychological factors. These factors and corresponding measures were carefully and comprehensively considered when developing the physical exercise evaluation system for children with asthma. Therefore, this physical exercise evaluation index system can be used as an evaluation tool for children with asthma and nursing staff can use the relevant indicators of this evaluation system to assess exercise conditions and provide targeted health education regarding physical exercise for children with asthma. Moreover, nurses and other health care professionals, such as doctors and respiratory rehabilitation personnel, can also use the index system to assess the physical exercise of children with asthma and develop effective care plans. Physical activity is an economical and healthy treatment strategy for asthma control. The application and promotion of this system can also help caregivers and patients attach importance to physical exercise, fully understand the relationship between asthma and exercise, and improve the management of asthma through safe and effective exercise programs.

### Limitations of the study

This study is an original index built on the basis of literature review and clinical internship, and a three-level evaluation index system formed after two rounds of Delphi expert letter consultation is still lacking in the actual application of indicators to the target population, and will be gradually carried out in the future.

## Conclusion

For children with asthma, exercise is a double-edged sword. Levels of physical activity that are too low or too high are related to poor asthma management effects and negative psychological reactions in children. A poor choice of exercise environment and exercise practices may aggravate asthma, preventing individuals from engaging in exercise. The physical exercise evaluation index system for school-aged children with asthma established in this study includes 4 primary indicators, 11 secondary indicators and 50 tertiary indicators, which comprehensively cover the exercise practices of asthmatic children as well as their influencing factors, such as disease factors, exercise environment factors, and exercise psychology factors. In addition, it is practical and operable. The evaluation index system can be used by children with asthma, their caregivers, and health care professionals to monitor the exercise practices of children with asthma and identify influential factors, which can lay the foundation for follow-up targeted interventions aimed at improving exercise practices.

## Supporting information

S1 Data The first Delphi. (XLSX)

S2 Data The second Delphi. (XLSX)

S3 Data The Ca Cs assignment support table. (XLSX)

S1 Table First letter questionnaire template. (DOCX)

S2 Table Second letter questionnaire template. (DOCX)
