# Supplementary material for: Establishment of a physical exercise evaluation index system for school-age children with asthma
Source: PLoS One. 2025 Jan 9;20(1):e0312398. doi: 10.1371/journal.pone.0312398 (PMC11717281; doi:10.1371/journal.pone.0312398)
Supplement: S1 Table — (DOCX) [file pone.0312398.s004.docx]

## Expert advice request letter

Dear expert,

Thank you for taking time out of your busy schedule to fill in the questionnaire "Construction of Physical Exercise Quality Evaluation Index System for school-age children with Asthma" and give evaluation opinions. Thank you for your support for this research!

1. Research background

Bronchial asthma is a chronic respiratory inflammatory disease, exercise is a common asthma trigger, but reasonable exercise can improve the management of asthma.In clinical practice, many patients lack of exercise-related knowledge, resulting in resistance to sports, reduced exercise, or unreasonable choice of disease conditions, exercise environment, exercise types, etc., which increases the risk of asthma.The prevalence rate of school-age children with asthma is high and they are at a critical stage of development. Therefore, the physical exercise quality evaluation index system for school-age children with asthma should be established to guide clinical patients to exercise safely and effectively.

2. Research purpose

To establish a physical exercise self-evaluation index system for school-age children with asthma, evaluate the safety and quality of physical exercise for school-age children with asthma, and promote the improvement of sports safety and quality.

3. The basis of construction

Based on the "know-believe-action" and "risk management theory" models, the exercise recommendations of the American College of Sports Medicine (ACSM) guidelines, the American Thoracic Society and the European Respiratory Society for children aged 6-17 years with asthma were followed.

This study was carried out in the form of expert correspondence.Please feedback your suggestions and comments by Jun 25, 2022. If you have any questions, please feel free to contact Researcher Yueheng Zhao. We will follow the principle of scientific research. The results of this questionnaire are only for scientific research purposes and will only release the research results. We will not disclose your privacy information to any organization or individual.

Thank you for your support and help!

# Establishment of a physical exercise self-evaluation index system for school-age children with asthma

（First round of expert consultation form）

Introduction of the system：

According to the literature review and clinical investigation, the related factors affecting the exercise quality of asthma patients were summarized, classified and analyzed one by one to form a pool of items.Finally formed a three-level rating index system, including: 4 first-level indicators, 13 second-level indicators, 39 third-level indicators.

Formfilling explanation：

This questionnaire is composed of four dimensions: disease, environment, knowledge and psychology. There are several items in each dimension. After each item, there is an importance evaluation column and a column of "Modification suggestions".

Each item is divided into 5= very important, 4= important, 3= generally important, 2= not very important, and 1= not important according to the degree of importance. In order to ensure the quality of the research, please check each indicator (indicated by "✓"), and all choices are single.

If you have any modification suggestions for the indicators, please fill in the column of "Modification Suggestions", and check the importance of the revised indicators. If you have different views on the composition of the scale dimensions or have other content to add, please fill in the column of "Opinions", and judge the importance of the indicators after adding or adjusting, do not omit or leave it blank.

Table 1 Relative importance of the first-level indicators

| First-level indicators | very important | important | generally important | not very important | not important | Modification Suggestions |
| --- | --- | --- | --- | --- | --- | --- |
|  | 5 | 4 | 3 | 2 | 1 |  |
| Disease Factors |  |  |  |  |  |  |
| Exercise environment |  |  |  |  |  |  |
| Exercise knowledge |  |  |  |  |  |  |
| Exercise Psychology |  |  |  |  |  |  |

Table 2 Relative importance of the second-level indicators

| First-level indicators | Second-level indicators | very important | important | generally important | not very important | not important | Modification Suggestions |
| --- | --- | --- | --- | --- | --- | --- | --- |
|  |  | 5 | 4 | 3 | 2 | 1 |  |
| Disease Factors | Disease Diagnosis |  |  |  |  |  |  |
|  | Asthma control level |  |  |  |  |  |  |
|  | Medication |  |  |  |  |  |  |
|  | First aid measures |  |  |  |  |  |  |
| Exercise environment | Natural environment |  |  |  |  |  |  |
|  | Custodial factors |  |  |  |  |  |  |
| Exercise knowledge | Exercise content |  |  |  |  |  |  |
|  | Exercise status |  |  |  |  |  |  |
|  | Exercise intensity |  |  |  |  |  |  |
|  | Exercise duration |  |  |  |  |  |  |
|  | Exercise frequency |  |  |  |  |  |  |
| Exercise Psychology | Positive psychology |  |  |  |  |  |  |
|  | Negative psychology |  |  |  |  |  |  |

Table 3 Relative importance of the third-level indicators

| First-level indicators | Second-level indicators | Third-level indicators | very important | important | generally important | not very important | not important | Modification Suggestions |
| --- | --- | --- | --- | --- | --- | --- | --- | --- |
|  |  |  | 5 | 4 | 3 | 2 | 1 |  |
| Disease Factors | Disease Diagnosis | Whether there are clear allergens |  |  |  |  |  |  |
|  |  | Whether motor asthma or motor bronchial spasm has been diagnosed |  |  |  |  |  |  |
|  | Asthma control level | Frequency of asthma symptoms during the day |  |  |  |  |  |  |
|  |  | No nighttime asthma symptoms or waking up with choking |  |  |  |  |  |  |
|  |  | Whether the activity is unrestricted |  |  |  |  |  |  |
|  |  | Frequency of drug use |  |  |  |  |  |  |
|  |  | Whether PEF/FEV1 is normal |  |  |  |  |  |  |
|  | Medication | Whether the role of the drug is known |  |  |  |  |  |  |
|  |  | Whether the correct medication method has been mastered |  |  |  |  |  |  |
|  |  | Whether compliance to the medication is high |  |  |  |  |  |  |
|  | First aid measures | Whether the signs of an impending asthma attack are recognized |  |  |  |  |  |  |
|  |  | Whether emergency asthma medication is carried |  |  |  |  |  |  |
|  |  | Whether the peak expiratory flow rate meter can be used correctly |  |  |  |  |  |  |
|  |  | Whether there is awareness of the self-care measures for acute attacks |  |  |  |  |  |  |
| Exercise environment | Natural environment | Whether the environment is warm |  |  |  |  |  |  |
|  |  | Whether the environment is moist |  |  |  |  |  |  |
|  |  | Whether the haze levels are low |  |  |  |  |  |  |
|  |  | Whether there are no allergens in the environment |  |  |  |  |  |  |
|  | Custodial factors | Whether an exhalation peak flow velocity meter is carried |  |  |  |  |  |  |
|  |  | Whether a heart rate monitor is worn |  |  |  |  |  |  |
|  |  | Whether there are guardians |  |  |  |  |  |  |
|  |  | Whether there is monitoring sports equipment |  |  |  |  |  |  |
| Exercise knowledge | Exercise content | Whether there are warm-up preparation activities (15 min) |  |  |  |  |  |  |
|  |  | Whether there is a combination of aerobic exercise, resistance exercise, and flexibility exercise |  |  |  |  |  |  |
|  |  | Whether pursed-lip breathing and abdominal breathing are performed |  |  |  |  |  |  |
|  | Exercise status | Whether exercise is avoided on an empty stomach and immediately after a meal |  |  |  |  |  |  |
|  |  | Whether the PEFR> 80% |  |  |  |  |  |  |
|  | Exercise intensity | Whether low-intensity movement is combined with moderate-intensity movement |  |  |  |  |  |  |
|  | Exercise duration | Whether the time is limited (20 ~ 60 min/d) |  |  |  |  |  |  |
|  | Exercise frequency | Whether regular exercise is performed an average of 3-5 days per week |  |  |  |  |  |  |
| Exercise Psychology | Positive psychology | Loves exercise or not |  |  |  |  |  |  |
|  |  | Whether the individual is convinced of the positive effects of exercise on asthma control |  |  |  |  |  |  |
|  |  | Whether there are guardians who provide support |  |  |  |  |  |  |
|  |  | Whether team movement is sought |  |  |  |  |  |  |
|  |  | Whether exercise is an enjoyable experience |  |  |  |  |  |  |
|  |  | Whether the individual can respond to exercise-induced asthma |  |  |  |  |  |  |
|  | Negative psychology | Whether there is fear of an asthma attack during exercise |  |  |  |  |  |  |
|  |  | Whether the individual is worried about exercise exacerbating asthma |  |  |  |  |  |  |
|  |  | Whether there is a psychological burden of worrying about asthma attacks |  |  |  |  |  |  |

If you have any other suggestions, please fill them in here:

Basic information questionnaire of experts

Table 4 Instructions for filling in the form: Please fill in the form according to your actual situation, or mark "√" in the corresponding field.

| name |  | sex |  | | age |  | education background |  |
| --- | --- | --- | --- | --- | --- | --- | --- | --- |
| degree |  | the title of a technical post |  | | title |  | telephone number |  |
| professional unit | |  | | | | | mail box |  |
| occupation（multiple choices） | | □clinic □nurse □scientific research □teaching□management | | | years of working | |  | |
| work domain | |  | | | | | | |
| Your familiarity with the research question | | □very familiar □familiar □General familiar □not very familiar □unfamiliar | | | | | | |
| How you judge the indicators | | reference for judging | | How much it affects your judgment（Mark "√" in the corresponding field） | | | | |
|  |  |  |  | large | | middle | small | |
|  |  | theoretical analysis | |  | |  |  | |
|  |  | practical experience | |  | |  |  | |
|  |  | academic resources | |  | |  |  | |
|  |  | personal feeling | |  | |  |  | |
| If there is any other evidence, please list | |  | |  | |  |  | |
|  |  |  | |  | |  |  | |
|  |  |  | |  | |  |  | |

Thank you for your support and help in this study. Thank you!
