# Supplementary material for: Establishment of a physical exercise evaluation index system for school-age children with asthma
Source: PLoS One. 2025 Jan 9;20(1):e0312398. doi: 10.1371/journal.pone.0312398 (PMC11717281; doi:10.1371/journal.pone.0312398)
Supplement: S2 Table — (DOCX) [file pone.0312398.s005.docx]

## Expert advice request letter

Dear expert,

Thank you for taking time out of your busy schedule to fill in the first round of consultation form of "Establishment of a physical exercise self-evaluation index system for school-age children with asthma".Thank you for your support for this research! Your guidance and suggestions have given the research group a good inspiration and help!After the first round of questionnaires were collected, the research group carefully considered the opinions and suggestions of each expert and organized the members of the research group to have a discussion. On this basis, we made a revision.The revised opinions of the first round of experts and the revised questionnaire are now provided to you for the second round of expert consultation.Please re-evaluate the consulting project with reference to the results of the first round of comprehensive evaluation. If you have different opinions on the evaluation results, please fill in the comments box.In addition, in the second round of consultation form, the research group added the weight consultation of each item of the physical exercise quality evaluation index system for school-age children with asthma, please fill in as required.Please feedback your suggestions and comments by November 25, 2022. If you have any questions, please feel free to contact Researcher Yueheng Zhao. We will follow the principle of scientific research. The results of this questionnaire are only for scientific research purposes and will only release the research results. We will not disclose your privacy information to any organization or individual.

Thank you for your support and help!

# Establishment of a physical exercise self-evaluation index system for school-age children with asthma

（Second round of expert consultation form）

Introduction of the system：

According to the first round of expert correspondence, the 3-level rating indicators in the system eventually formed 4 first-level indicators, 11 second-level indicators, and 50 third-level indicators.

Formfilling explanation：

Each item is divided into 5= very important, 4= important, 3= generally important, 2= not very important, and 1= not important according to the degree of importance. In order to ensure the quality of the research, please check each indicator (indicated by "✓"), and all choices are single.

If you have any modification suggestions for the indicators, please fill in the column of "Modification Suggestions", and check the importance of the revised indicators. If you have different views on the composition of the scale dimensions or have other content to add, please fill in the column of "Opinions", and judge the importance of the indicators after adding or adjusting, do not omit or leave it blank.

Table 1 Relative importance of the first-level indicators

| First-level indicators | very important | important | generally important | not very important | not important | Modification Suggestions |
| --- | --- | --- | --- | --- | --- | --- |
|  | 5 | 4 | 3 | 2 | 1 |  |
| Disease Factors |  |  |  |  |  |  |
| Exercise environment |  |  |  |  |  |  |
| Exercise Practice |  |  |  |  |  |  |
| Exercise Psychology |  |  |  |  |  |  |

Table 2 Relative importance of the second-level indicators

| First-level indicators | Second-level indicators | very important | important | generally important | not very important | not important | Modification Suggestions |
| --- | --- | --- | --- | --- | --- | --- | --- |
|  |  | 5 | 4 | 3 | 2 | 1 |  |
| Disease Factors | Disease Diagnosis |  |  |  |  |  |  |
|  | Asthma control level |  |  |  |  |  |  |
|  | Medication |  |  |  |  |  |  |
|  | First aid knowledge and skills |  |  |  |  |  |  |
| Exercise environment | Natural environment |  |  |  |  |  |  |
|  | Custodial factors |  |  |  |  |  |  |
| Exercise Practice | Exercise items |  |  |  |  |  |  |
|  | Exercise status |  |  |  |  |  |  |
|  | Exercise plan |  |  |  |  |  |  |
| Exercise Psychology | Positive psychology |  |  |  |  |  |  |
|  | Negative psychology |  |  |  |  |  |  |

Table 3 Relative importance of the third-level indicators

| First-level indicators | Second-level indicators | Third-level indicators | very important | important | generally important | not very important | not important | Modification Suggestions |
| --- | --- | --- | --- | --- | --- | --- | --- | --- |
|  |  |  | 5 | 4 | 3 | 2 | 1 |  |
| A  Disease Factors | A1 Disease Diagnosis | A1.1 Whether there are clear allergens |  |  |  |  |  |  |
|  |  | A1.2 Whether motor asthma or motor bronchial spasm has been diagnosed |  |  |  |  |  |  |
|  |  | A1.3 Whether there are clear triggers |  |  |  |  |  |  |
|  |  | A1.4 Whether there are any other coexisting respiratory system diseases |  |  |  |  |  |  |
|  | A2 Asthma control level | A2.1 Whether the frequency of daytime asthma symptoms is ≤ 2 times/week |  |  |  |  |  |  |
|  |  | A2.2 No nighttime asthma symptoms or waking up with choking |  |  |  |  |  |  |
|  |  | A2.3 Whether the activity is unrestricted |  |  |  |  |  |  |
|  |  | A2.4 Whether alleviating drugs or first aid treatment is needed |  |  |  |  |  |  |
|  |  | A2.5 Whether the FEV1 value is > 80% |  |  |  |  |  |  |
|  | A3 Medication | A3.1 Whether the main role of the drug used is known |  |  |  |  |  |  |
|  |  | A3.2 Whether how to use the drugs is known |  |  |  |  |  |  |
|  |  | A3.3 Whether the time, frequency and contraindications of oral medication are known |  |  |  |  |  |  |
|  |  | A3.4 Whether the preparation and use of inhalation devices have been mastered |  |  |  |  |  |  |
|  |  | A3.5 Whether the ability to use inhalation drugs has been mastered |  |  |  |  |  |  |
|  |  | A3.6 Whether the physician-directed regular quantitative medication schedule is followed |  |  |  |  |  |  |
|  | A4 First aid knowledge and skills | A4.1 Whether the signs of an impending asthma attack are recognized |  |  |  |  |  |  |
|  |  | A4.2 Whether the signs of an acute asthma exacerbation can be identified |  |  |  |  |  |  |
|  |  | A4.3 Whether emergency asthma medication is carried |  |  |  |  |  |  |
|  |  | A4.4 Whether the peak expiratory flow rate meter can be used correctly |  |  |  |  |  |  |
|  |  | A4.5 Whether there is awareness of the self-care measures for acute attacks |  |  |  |  |  |  |
| B  Exercise  environment | B1 Natural environment | B1.1 Whether the temperature is considered and whether the time when the temperature is comfortable is chosen |  |  |  |  |  |  |
|  |  | B1.2 Whether the humidity is considered and whether the time when the humidity is comfortable is chosen |  |  |  |  |  |  |
|  |  | B1.3 Whether attention is paid to the degree of haze as identified using mobile phones and whether outdoor sports are chosen only when the level is low |  |  |  |  |  |  |
|  |  | B1.4 Whether the exercise environment is evaluated for allergens |  |  |  |  |  |  |
|  | B2 Custodial factors | B2.1 Whether the exhalation peak flow velocity instrument is carried to predict an acute attack |  |  |  |  |  |  |
|  |  | B2.2 Whether a heart rate monitor is worn to monitor activity |  |  |  |  |  |  |
|  |  | B2.3 Whether there are adult guardians present during exercise |  |  |  |  |  |  |
|  |  | B2.4 Whether treadmills that can monitor exercise time and intensity-monitoring equipment are used |  |  |  |  |  |  |
| C  Exercise  Practice | C1 Exercise items | C1.1 Whether there are warm-up preparation activities (15 min) |  |  |  |  |  |  |
|  |  | C1.2 Whether there is a cooldown activity after exercising (15 min) |  |  |  |  |  |  |
|  |  | C1.3 Whether there is a combination of aerobic exercise, resistance exercise, and flexibility exercise |  |  |  |  |  |  |
|  |  | C1.4 Whether pursed-lip breathing and abdominal breathing are performed |  |  |  |  |  |  |
|  | C2 Exercise status | C2.1 Whether exercise is avoided on an empty stomach and immediately after a meal |  |  |  |  |  |  |
|  |  | C2.2 Whether general physical activity causes difficulty breathing |  |  |  |  |  |  |
|  |  | C2.3 Whether the heartbeat is normal without chest pain |  |  |  |  |  |  |
|  | C3 Exercise plan | C3.1 Whether low-intensity movement is combined with moderate-intensity movement |  |  |  |  |  |  |
|  |  | C3.2 Whether an exercise schedule is made in advance |  |  |  |  |  |  |
|  |  | C3.3 Whether exercise logs are recorded after exercise |  |  |  |  |  |  |
|  |  | C3.4 Whether the time is limited (20 ~ 60 min/d) |  |  |  |  |  |  |
|  |  | C3.5 Whether regular exercise is performed an average of 3-5 days per week |  |  |  |  |  |  |
| D  Exercise  Psychology | D1 Positive psychology | D1.1 Loves exercise or not |  |  |  |  |  |  |
|  |  | D1.2 Whether the individual is convinced of the positive effects of exercise on asthma control |  |  |  |  |  |  |
|  |  | D1.3 Whether the guardian’s opinion is accepted regarding exercise |  |  |  |  |  |  |
|  |  | D1.4 Whether exercise plans are considered favorable |  |  |  |  |  |  |
|  |  | D1.5 Whether there is confidence in adhering to the exercise plan |  |  |  |  |  |  |
|  |  | D1.6 Whether the individual participates in collective movement happily |  |  |  |  |  |  |
|  |  | D1.7 Whether exercise is an enjoyable experience |  |  |  |  |  |  |
|  |  | D1.8 Whether the individual can respond to exercise-induced asthma |  |  |  |  |  |  |
|  | D2 Negative psychology | D2.1 Whether there is fear of an asthma attack during exercise |  |  |  |  |  |  |
|  |  | D2.2 Whether there are worries about exercise affecting the treatment effect |  |  |  |  |  |  |

If you have any other suggestions, please fill them in here:

Basic information questionnaire of experts

Table 4 Instructions for filling in the form: Please fill in the form according to your actual situation, or mark "√" in the corresponding field.

| name |  | sex |  | | age |  | education background |  |
| --- | --- | --- | --- | --- | --- | --- | --- | --- |
| degree |  | the title of a technical post |  | | title |  | telephone number |  |
| professional unit | |  | | | | | mail box |  |
| occupation（multiple choices） | | □clinic □nurse □scientific research □teaching□management | | | years of working | |  | |
| work domain | |  | | | | | | |
| Your familiarity with the research question | | □very familiar □familiar □General familiar □not very familiar □unfamiliar | | | | | | |
| How you judge the indicators | | reference for judging | | How much it affects your judgment（Mark "√" in the corresponding field） | | | | |
|  |  |  |  | large | | middle | small | |
|  |  | theoretical analysis | |  | |  |  | |
|  |  | practical experience | |  | |  |  | |
|  |  | academic resources | |  | |  |  | |
|  |  | personal feeling | |  | |  |  | |
| If there is any other evidence, please list | |  | |  | |  |  | |
|  |  |  | |  | |  |  | |
|  |  |  | |  | |  |  | |

Thank you for your support and help in this study. Thank you!
